# Parent’s food preference and its implication for child malnutrition in Dabat health and demographic surveillance system; community-based survey using multinomial logistic regression model: North West Ethiopia; December 2017

**DOI:** 10.1186/s12887-019-1692-3

**Published:** 2019-09-02

**Authors:** Nigusie Birhan Tebeje, Gashaw Andargie Biks, Solomon Mekonnen Abebe, Melike Endris Yesuf

**Affiliations:** 1 School of Nursing, College of Medicine and Health Sciences, University of Gondar, Gondar, Ethiopia; 2 Department of Health Service Management and Health Economics, Institute of Public Health, College of Medicine and Health Sciences, University of Gondar, Gondar, Ethiopia; 3 Department of Human Nutrition, Institute of Public Health, College of Medicine and Health Sciences, University of Gondar, Gondar, Ethiopia

**Keywords:** Under-five, Children, Food preference, Dabat, Parent, Caretaker

## Abstract

**Background:**

A Shortage or excessive intake of the nutrient is malnutrition; affecting every aspect of human beings. Malnutrition at childhood has long-lasting and multiple effects. In Ethiopia significant numbers of children were suffering from malnutrition that might be associated with parents’ food preference; the fact not yet investigated. Therefore the aim of this study was to assess parents’ food preferences and its implication for child malnutrition.

**Methods:**

The study was conducted among 7150 mothers/caretakers in Dabat demographic and health surveillance site. Data were collected by experienced data collectors working for the surveillance centers after extensive training. A multinomial logistic regression model was fitted to determine the effect of factors on the dependent variable and model fitness was checked using a likelihood ratio test.

**Results:**

About 62.55% of mothers/caretakers prefer to feed children with a family and 16.45% of them prefer to feed children with a specific type of food. Mothers/caretakers who introduce semisolid food after 6 months 2.34(1.50–3.96) were times more likely prefer to feed with family food for their children than a balanced diet. Regarding the specific type of food preference mothers who introduce semisolid food after 6 months and those obtain food from the market were 6.53(3.80–11.24) and 4.38(3.45–5.56) times more likely to prefer to feed specific types of than balanced diet respectively.

**Conclusion:**

Food preference had contributed to the increased and persistent magnitude of child malnutrition as 62.55% of mothers prefer to feed children with family and only 21% of them prefer to feed a balanced diet for under-five children. Therefore we recommended integration of child dietary diversity, acceptability and safety counseling session for mothers visiting health institutions for child vaccination, ANC and PNC services.

**Electronic supplementary material:**

The online version of this article (10.1186/s12887-019-1692-3) contains supplementary material, which is available to authorized users.

## Background

Malnutrition is a failure of the body to get an appropriate amount of nutrients for healthy human organ and tissue function. Children were more vulnerable to malnutrition. Children who suffer from nutritional deprivation were at risk of developmental delays which can lead to different consequences [1]. In the year 2007, the Lancet estimated that about 200 million under-five children were failing to fulfill developmental potential in developing countries due to malnutrition [2]. According to the MDG report in 2012 malnourished children at adulthood are estimated to earn 20% less than their counterparts [3]. The young lives survey in its 2010 report in developing countries suggests that by of age 7 or 8 years older the malnutrition consequence is comparable to a loss of full-term schooling and is associated with the loss of 10–15 IQ points [4, 5].

The global burden of diseases suggested that underweight in young children is one of the leading cause of burden of disease in sub-Saharan Africa. It is responsible for increased years of lives with a disability for children under 5 years [5]. In 2013 almost 6.3 million children under 5 years lost their life from preventable causes and every year about 2.6 million under-five children died because of malnutrition [6].

In the year 2011 10 years after setting the goal of eradicating extreme hunger globally about 314, 258, and 52 million children below the age of five were suffering from stunting, underweight and wasting respectively [7]. Malnutrition occurring in the first 1000 days of life has long-lasting irreversible consequence including being stunting forever, susceptible to sickness, poor school performance, entering adulthood more likely to become overweight and prone to none communicable disease [8].

Malnutrition is a priority problem since the 1970s but not addressed yet because it may be related to mothers/caretakers food preference uninvestigated fact but have potential to affect safety, diversity, acceptability, and frequency of food basic dimensions for good nourishment of children [9]. Another nutrition-related emerging public health problem more prominently related to food preference is an increased rate of overweight and expected to nearly double again by 2025 but not yet investigated well in middle and low-income countries [10].

It is agreed on the fact that no child is born to die from the cycle of malnutrition and our world is believed to have enough food for every one of us [3]. However, currently available evidence on child malnutrition was limited to determine the prevalence of malnutrition and revealed that 40% of under-five children in the globe were experiencing hunger. On the contrary works in FAO shows that world agriculture can produce enough to feed humanity indicating that there is an uninvestigated fact that probably related to parental food preference. We hypothesize that mothers/caretakers food preference may be the main contributor for child malnutrition which negatively interacting-with quality, diversity, frequency, safety, acceptability, and quantity of food in addition to ensuring food security and healthcare [11, 12]. Therefore this study was intended to generate information on the parent/caretakers food preference and its implication for child malnutrition in Dabat health and demographic site for national, regional and local decision-makers.

## Methods

### Study area

**Fig. 1:**
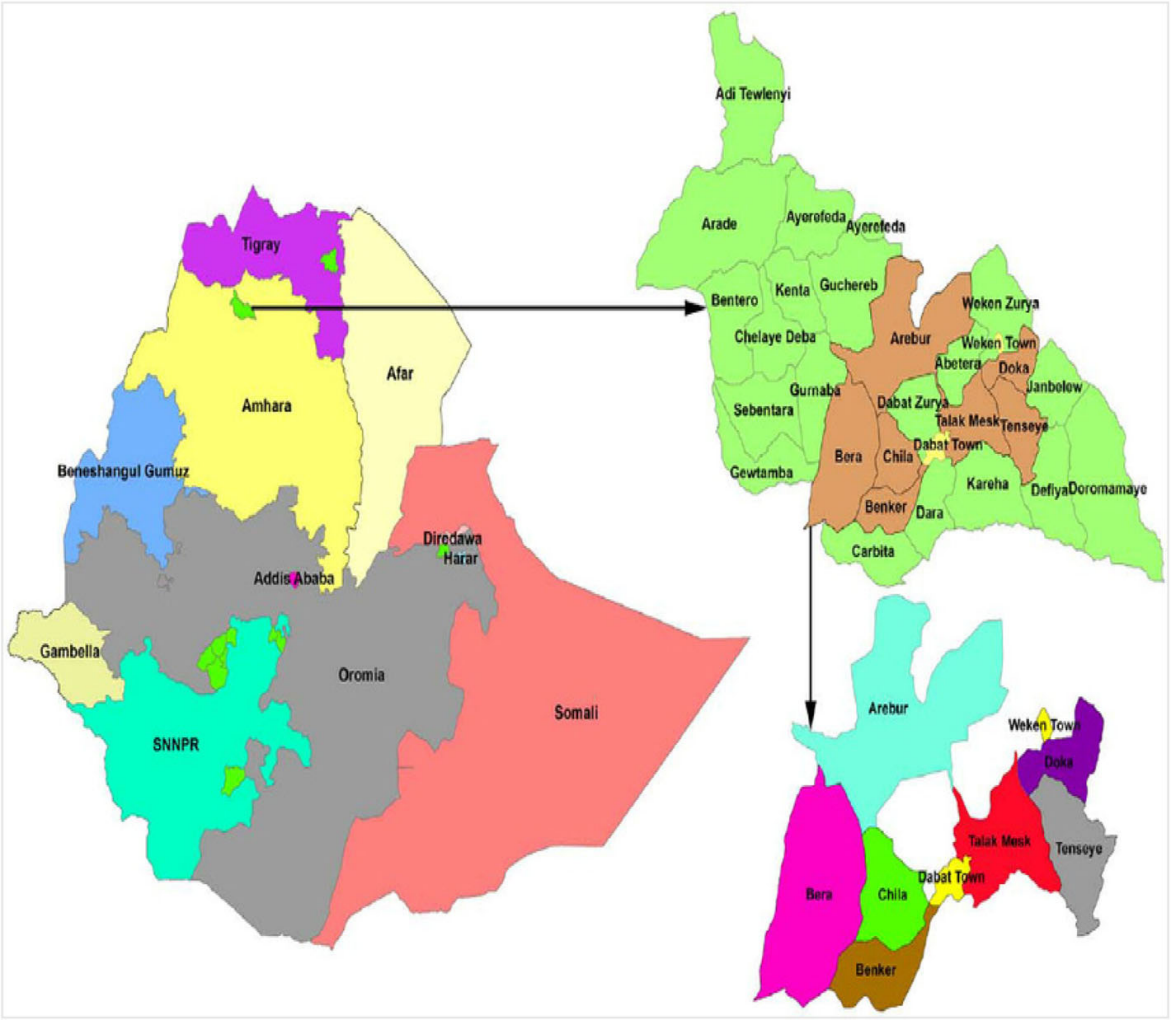
The figure showing the maps of the nation, the region, the district and the kebeles included in the survey uploaded by Almayehu Worku available at http://www.biomedcentral.com/1471–2458/13/168

Study was conducted in Dabat district among 13 kebeles included in Dabat Demographic and Health Surveillance system site (DHSS) (Fig. 1). The altitude of the HDSS is divided into high land, Midland, and low land climatic conditions. According to the Woreda health office reports, the district has six health centers, three health stations, and thirty-one health posts that provide health services to the community. The total population of the district was estimated to be 158, 250 of whom 70, 611 people were the population of the HDSS with almost 1:1 sex ratio. The DHSS has 7918 children under the age of 5 years from 6314 households [13].

**Study design and population:** the community-based cross-sectional study was carried out among rural and urban households from April to December 2016. Mothers /caretakers with under-five children (6–59 months) and found in the HDSS were the study participants.

**Data collection tool and data collection procedure:** A pre-tested interviewer-administered structured questionnaire developed by the investigators in English language translated to local language was used to collect data on socio-demographic, health characteristics, child feeding characteristics and food preference habits of mothers /caretakers of the under-five children (Additional file 1). A five-day intensive training was provided for data collectors and supervisors. A pre-test was conducted in the rural and urban kebeles which are not included in the HDSS. The necessary modification was made on the tool according to the inputs obtained from the pre-test. Data were collected by 15 experienced data collectors and supervised by 5 supervisors working for Dabat HDSS.

**Data processing and analysis:** Data were entered into Epi data template prepared by the Amharic language to avoid data entry errors by five experienced data entry clerks working for Dabat HDSS. The data entry process was supervised by the data manager working for the HDSS. Entered data were transported to STATA version 12 for further analysis. Before the actual data analysis, data clearance was performed. After data clearance and recoding, a multinomial logistic regression model was fitted to identify predictors for mothers/ caretakers preference to feed specific type of food, family food or balanced diet for their under-five children.

**Dependent variable:** Mothers/caretakers food preference for under-5 year’s children.

### Independent variables

**Socio-demographic characters: -** (age and sex of the child, birth order and interval of the child, maternal educational status, parents educational status, family size, religion ethnicity, occupation).

**Environmental factor: -** (means of transportation, the distance of the market, food item buying habits and frequency, residence).

**Health factors:-** (child illness, PNC, ANC utilization, child immunization status).

### Operational definition

**Food preference:** If parents choose to feed food with the same caloric content more than once per day it is considered as preferring to feed specific food preference, if they tend to feed any available food or the food prepared for adult family members it is considered as a preference to feed family food and if there is a habit of balancing child food from locally available food items it is a preference to feed a balanced diet.

## Result

About 6896 participants were willing to respond for the interview making the response rate of 97.4%. Almost half (50.5%) of children were female. More than three-fourths (79.86%) and two-thirds (68.00%) of mothers /caretakers were rural residents and farmers by occupation. Majority of mothers/caregivers (81.20%) were Orthodox Christians and 86.29% were currently married. A large proportion (74.23%) of households with under-five children had a garden to grow cereals and grains (Table 1).

**Table 1:** Socio-demographic characteristics and feeding practice of under-five children in Dabat health and demographic surveillance system: Dabat district North West Ethiopia 2017

Variable	Category	Frequency	Percentage	Remark
Sex	Male	3413	49.50	
	Female	3483	50.50	
Age	6–12 months	1025	14.95	
	13–18 months	728	10.43	
	19–24 months	794	11.54	
	25–30 months	726	10.39	
	31–36 months	809	11.74	
	37–42 months	657	9.57	
	43–50 months	2157	31.39	
Birth order	First birth	1322	19.17	
	Second birth	1, 141	16.54	
	Third birth	1, 055	15.29	
	Fourth birth	1021	14.82	
	Fifth and above	2357	34.17	
Birth interval	One year	235	3.41	
	Two years	1429	20.72	
	Three years	2605	37.78	
	Fourth years	1229	17.82	
	Five year	1398	20.27	
Source of food items	Garden	5119	74.23	
	Market	1777	25.77	
Available food items	Fruit and vegetables	2276	32.53	
	All types of meat	5432	77.63	
	Egg and milk	4750	67.89	
	Cereal and grains	6797	97.14	
	Root and tubers	247	3.53	
Relation of caregivers	Mother	6589	95.55	
	Grandmother	218	3.15	
	Father and other relatives	89	2.22	
Preparation of child food	Separately for children	1624	23.55	
	With adults	5272	76.45	
Child feeding practice	Alone	4205	60.97	
	With older children	967	14.02	
	With adults	1678	24.33	
	Before adults and older children	9	0.13	
	After adults and older children	37	0.54	
a distance of the nearby market	1–4 km	1350	75.99	
	5–10 km	49	2.79	
	11–20 km	109	6.11	
	≥21 km	269	15.10	
Frequency of food buying	Daily	45	2	
	2–3 times per week	172	7.6	
	Weekly	574	25.3	
	One in two weeks	332	14.66	
	Once per month	1022	45.1	
	Once in four months	122	5.4	
Means of transportation to market	Foot	1770	99.60	
	Public transport	7	0.40	
Maternal education	Unable to read and write	4794	69.52	
	Primary education	1406	20.39	
	Secondary and above	696	10.09	
Residence	Rural	5507	79.86	
	Urban	1389	20.14	
Ethnicity	Amhara	5470	79.33	
	Tigery	1176	17.05	
	Others	250	3.62	
Religion	Orthodox	5599	81.20	
	Muslim	1176	17.05	
	Others	121	1.75	
Maternal Occupation	Farmer	4689	68.00	
	Merchant/employed	234	3.39	
	Housewife	1514	21.96	
	Others	459	6.65	
Marital status	Married	5950	86.29	
	No married	638	9.24	
	Separated /divorced	308	4.47	

### Mothers /caretakers food preference and feeding practice in Dabat district

From the total 4313 (62.55%) of mothers/caretakers prefers feed with the portion of family food and 1135(16.45%) of them prefers to feed their under-five children with a specific type of food more than once per day. Regarding balancing of child food from locally available food items 1448 (21%) of mothers/ caretakers prefer to feed a balanced diet food for under-five children (Table 2).

**Table 2:** Distribution of mothers/caretakers food preference with socio-demographic attributes: Dabat HDSS North West Ethiopia, 2017

Variables	Food/feeding preference			
	No preference/family food	Specific food preference	Balanced diet	Total
Age				
First year	581	199	227	1007
Second year	945	285	307	1537
Third year	1000	248	303	1551
Fourth year	1155	275	375	1805
Fifth year	676	137	250	1063
Total	4357	1144	1462	6963
Sex				
Male	2151	569	724	3444
Female	2212	578	735	3525
Total	4363	1147	1459	6969
Birth order				
First order	789	272	286	1347
Second order	701	207	236	1144
Third order	670	164	220	1054
Fourth order	649	149	221	1019
Five & above	1531	344	500	2375
Total	4340	1136	1463	6939
Introduction of supplementary food				
< 6 months	115	109	313	537
At six month	688	876	2489	4053
7–11 months	182	179	634	995
At one year	121	268	747	1136
After one year	30	27	129	186
uncertain	7	0	37	44
Total	1143	1459	4349	6951
ANC visit				
one &two visit	682	163	239	1084
three visits	1001	251	388	1640
four visits	761	271	297	1329
Five &above	265	129	77	471
No ANC visit	1623	316	452	2391
Total	4332	1130	1453	6915

### Factors associated with food preference among parents of under-five children Dabat HDSS

Among variables entered in to univariate multinomial logistic regression maternal religion, maternal inability to read and write 2.19(1.09–4.40), introducing semisolid food after six months 1.10 (1.02–1.16), feeding child once in 24 h CORRR = 2.65(CI = 1.52–4.62), child age of 25–36 months CORRR = 1.29(CI = 1.05–1.57), one ANC visit during pregnancy CORRR = 2.07 (CI = 1.39–3.07) were associated with increased odds of preferring family food for the child. While attending ANC in hospital CORR = 3.44 (CI = 1.61–7.37) obtaining food from market CORR = 4.23(CI = 3.47–5.14) and having five and above ANC visit during pregnancy CORR = 1.83(CI = 1.30–2.58) were associated with increased odds of preferring a specific type of food for the children.

As shown in Table 3 maternal inability to read and write ARRR = 2.19(CI = 1.09–4.40), introducing semisolid food after 6 months ARRR = 2.34(CI = 1.50–3.96), and residing more than 4kms from a local market ARRR = 2.41(CI = 1.97–2.96) were associated with increased odds of preferring to feed a child with the family food. Similarly introducing semisolid food after 6 months 6.53(3.8–11.24), and obtain food from market ARRR = 4.38 (CI = 3.45–5.56) were associated with the increased odds of preferring to feed specific type of food for the children (Table 3).

**Table 3:** Multinomial logistic regression table showing factors associated with parents/caretakers food preference to feed under-five year’s children in Dabat HDSS; Dabat district northwest Ethiopia: 2017

	Base outcome balanced diet preference					
Predictor /variable	Family food preference			Specific type of food		
Religion	Number	CRRR(95%CI)	ARRR(95%CI)	Number	CRRR(95%CI)	ARRR(95%CI)
Orthodox	3094	1.00	1.00	759	1.00	1.00
Muslim	473	0.84(0.7–1.01)	0.54(0.11–2.82)	166	1.20(0.95–1.51)	0.67(011–4.53)
Others	58	2.40(1.12 5.02)*	0.45(0.03–5.37)	39	6.55(3.04–14.11)	1.23(0.07–20.66)
Maternal EDU						
Unable to read & write	1632	1.60(1.20–2.08)	2.19(1.09–4.40)**	453	0.08(0.58–1.10)	1.24(0.53–2.89)
Primary EDU	445	1.30(0.94–1.77)	1.42(0.69–2.92)	130	0.70(0.48–1.10)	0.88(0.37–2.14)
Secondary+	182	1.00	1.00	99	1.00	1.00
Occupation						
Farmer	1592	1.00	1.00	474	1.00	1.00
Merchant	30	0.47(0.26–0.85)	0.88(0.25–3.17)	4	0.21(0.07–0.63)*	0.32(0.05–2.06)
Employed	37	2.23(0.87–5.70)	6.53(0.83–51.60)	26	5.25(2.00–13.8)*	4.80(0.51–44.89)
House wife	469	0.81(0.66–0.99)	0.71(0.39–1.28)	117	0.68(0.52–0.88)*	0.64(0.29–1.38)
Others	131	0.76(0.54–1.06)	1.22(0.55–2.72)	61	1.18(0.80–1.75)	1.08(0.41–2.89)
Period of excusive BF	4287	0.99(0.93–1.06)	0.42(0.26–0.66)**	1113	0.70(0.64–0.76)*	0.13(0.26–0.66)
Period of breast feeding	2060	0.89(0.78–1.01)	0.80(0.64–0.96)**	526	0.72(0.62–0.85)*	0.66(0.07–0.84)**
Age at intr.of food	4345	1.10(1.02–1.16)*	2.34(1.50–3.96)**	11,138	0.90(0.83–0.98)*	6.53(3.8–11.24)**
Frequency of feeding per 24 h						
Zero times	91	0.92(0.63–1.36)	0.76(0.19–3.05)	32	1.07(0. .66–1.75)	0.80(0.21–3.13)
One	98	2.65(1.52–4.62)*	2.82(0.77–10.36)	37	3.32(1.79–6.14)*	3.52(0.98–12.61)
Twice	351	1.37(1.07–1.75)*	1.71(0.76–3.85)	115	1.48(1.10–2.00)*	2.15(0.97–4.75)
Three time	1307	0.88(0.76–1.02)	0.99(0.62–1.60)	328	0.73(0.60–0.88)*	1.10(0.69–1.75)
Four time	1152	1.00	1.00	348	1.00	1.00
Five and above	1354	2.38(1.20–2.84)*	1.38(0.80–2.38)	283	1.65(1.32–2.05)*	1.46(0 .85–2.47)
Birth order						
First	792	1.00	1.00	271	1.00	1.00
Second	699	1.07(0.88–1.31)	1.63(0.24–11.25)	207	0.93(0.72–1.91)	1.81(0.13–25.33)
Third	671	1.09(0.89–1.34)	1.38–0.20-9.54	164	0.78(0.60–1.01)*	1.04(.07–14.79)
Fourth	647	1.07(0.87–1.34)	0.92(0.13–6.43)	149	0.72(0.55–0.94) *	0.78(0.05–11.1)
Fifth	564	0.99(0.81–1.23)	1.16(0.16–8.11)	126	0.65(0.49–0.86) *	0.57(0.04–8.29)
Six and above	975	1.17(0.97–1.41)	1.75(0.25–12.18)	218	0.76(0.60–0.97) *	1.4(0.09–19.68)
Age of the child						
6–12 months	581	1.00	1.00	199	1.00	1.00
13–24 months	925	1.13(0.91–1.39)	0.81(0.44–1.51)	285	0.98(0.75–1.27)	0.81(0.43–1.51)
25–36 months	1000	1.29(1.05–1.57)*	0.83(0.44–1.56)	248	0.93(0.93–1.20)	0.83(0.44–1.56)
37–48 months	1060	1.20(0.98–1.45)	0.68(0.35–1.30)	252	0.83(0.64–1.06)	0.68(0.35–1.30)
49–60 months	771	1.08(0.88–1.33)	1.19(0.56–2.52)	160	0.65(0.50–0 .86)*	1.19(0. 56–2.52)
TT vaccination during pregnancy						
Yes	2150	0.69(0.41–1.18)	1.36(0.42–4.36)	623	0.56(0.30–1.05)	1.36(0.42–4.36)
No	486	0.43(0. .25–0.75)*	1.03(0.31–3.47)	174	0.43(0.22–0.83)*	1.03(0.31–3.47)
I don’t know	73	1.00	1.00	26	1.00	1.00
Iron tablet supplementation during pregnancy						
Yes	2392	0.88(0.69–1.11)	1.16(0.67–2.03)	708	0.73(0.54–0.96)*	1.16(0.67–2.03)
No	311	1.00	1.00	112	1.00	1.00
ANC Visit during pregnancy						
No visit	1656	1.42(1.20–1.68)*	0.99(0.63–1.57)	331	0.80(0.64–0.99) *	0.88(0.48–1.58)
One visit	173	2.07(1.39–3.07)*	1.47(0.61–3.55)	37	1.25(0.76–205)	0.82(0.25–2.67)
two visits	512	0.98(0.79–1.21)	0.99(0.57–1.72)	126	0.68(0.51–0.90) *	0.89(0.43–1.85)
three visits	999	1.02(0.86–1.22)	0.67(0.47–1.24)	253	0.73(0.58–0.92) *	0.96(0.52–1.79)
four visits	762	1.00	1.00	270	1.00	1.00
Five and above	240	1.39(1.03–1.88)	0.82(0.37–1.75)	112	1.83(1.30–2.58) *	1.25(0.48–3.25)
Place of ANC visit during pregnancy						
Health center	2349	0.63(0.49–0.82)*	1.25(0.43–3.67)	699	0.70(0.50–0.96)*	1.25(0.43–3.67)
Health post	318	1.00	1.00	86	1.00	1.00
Hospital	35	0.86(0.41–1.81)	2.84(0.65–12.32)	338	3.44(1.61–7.37)*	2.84(0.65–12.32)
Birth interval						
One year	125	1.03(0.68–1.54)	0.59(0.20–1.69)	30	1.60(0.62–1.08)	0.59(0.15–2.36)
Two years	764	1.00	1.00	178	1.00	1.00
Three years	1282	0.69(0.58–0.83)*	0.55(0.37–0.81)**	312	0.72(0.57–0.92) *	0.64(0. 39–1.05)
Four years	628	0.78(0.63–0.97)*	0.73(0.45–1.18)	130	0.69(0.52–0.93) *	0.61(0.32–1.15)
Five and above	730	1.07(0.86–1.33)	1.03(0.60–1.75)	203	1.27(0.96–1.96)	0.78(0.40–1.54)
Obtaining food items from garden						
Yes	2968	1.00	1.00	625	1.00	1.00
No	1047	2.19(1.86–2.59)*	2.41(1.97–2.96)**	424	4.23(3.47–5.14)*	4.38(3.45–5.56)**
Frequency of buying food items						
Daily	24	1.00	0.75(0. 23–2.41)	17	1.00	1.07(0.32–3.55)
2–3 per week	76	0.37(0.12–1.15)	0.36(0.18–0.61)**	59	0 .41(0.13–1.31)	0.39(0.21–0 .74)**
Weekly	330	1.02(0.34–3.05)	0.57(0.35–0.94)**	184	0.80(0.26–2.48)	0.88(0.52–1.48)
In two weeks	201	0.44(0. 15–1.31)	0.23(0.14–0.38)**	53	0.16(.05–0.51)*	0.21(0.12–0.38)**
Monthly	739	1.81(0.61–5.37)	1.00	203	0.70(0.23–2.16)	1.00
> a month	88	1.46(0.42–5.08)	0.67(0.26–1.76)	23	0.54(0.14–2.02)	0.75(0.27–2.10)
Distance to local market	1465	1.57(1.39–1.78)*	1.41(1.17–1.70)**	541	1.09(0. 95–1.25)	0.96(0.77–1.20)

* Significant at univariate model with *p*-value < 0.005

**significant at multivariate model with p-value < 0.00s

*EDU* educational status

*Intro* introduction

## Discussion

Diversification and balancing of food are the strategies to address the nutritional problem of children. In this study, only 21% of mothers/caretakers prefer to feed a balanced diet, 62.55% of prefers to feed family food and 16.45% prefers to feed specific type of food for children. Preferring to feed children with family and specific type of food imply child malnutrition as it harms dietary diversity and dietary frequency contributors for child malnutrition [14, 15]. This explanation was supported by evidence that reported the possibility of reducing the odds of stunting with increased dietary diversity [16–21]. In our study area, child malnutrition is a major problem where 40, 9, 25% of children were stunted wasted and underweight respectively that may be mainly attributed by inappropriate food preference by mothers/caretakers evidenced by the result of this study [22].

In this study area, about 68% of participants were farmers who have two possible options to feed their under-five children. The first option is feeding children as adult members in the morning and at night, difficult to attain minimum acceptable food diversity and frequency issues strongly associated with increased odds of child malnutrition [18, 23]. The second option would be a takeover of cooked food to the farmland and feeding the child the whole day the takeover food. These options have to be questioned against its safety which worsens their health condition another issue which has strong implication child malnutrition [23–28].

Mothers/caretakers who were unable to read and write, introduce semisolid food after 6 months and walk more than 4kms to market were 2.19(1.09–4.40), 2.34(1.50–3.96) and 1.41(1.17–1.70) times respectively more likely to prefer to feed their under-five children with a family food than balanced diet in this study. The association between the above three factors and feeding a child with a family food may be explained by the fact that those unable to read and write, introduce semisolid food before 6 months and walk more than 4kms to the market to obtain food would be unable to comply with appropriate child feeding recommendations due to the inaccessibility of health, nutritional or child food conditions which have implication for child malnutrition by interfering with safety, diversity, and frequency of child food [28].

Similarly, mothers/caretakers who introduce semisolid food after 6 months were and obtain food items from the market were 6.53(3.8–11.24) and 4.38(3.45–5.56) times more likely to feed specific type of food for under-five children than feeding with a balanced diet. The association of late introduction of semi-fluid food and preference to feed a child with a specific type of food may be due to miss understanding of child feeding practice as the main reason of preference to feed a specific type of food for about 53% of the participants in this study was improving child health. Similarly, positive association between walking a far distance to the market and preference feed a specific type of food may be due to the difficulty of buying diversified food frequently as almost all those who buy food in this study walks on foot to the market. Such specific food preference for any reason has a contribution for child malnutrition as it has a direct effect on reduced diversity of the child food evidenced Chinese study that showed to a reduced score of height for weight with reduced dietary diversity [29].

On the other hand, exclusively breastfeed a child for 6 months 58% (34–74%), breastfeed for 2 years 20%(4–36%) and having 3 years birth interval between births 45%(19–63%) were associated with a decreased odds of preferring to feed a child with family food. In all of the above cases, mothers/caretakers may be better informed about appropriate child feeding practice and family planning service strategies to address child malnutrition [30].

Continuing breastfeeding for 2 years 79%(62–88) and buy food in 2 weeks frequency 34%(16–93%) were also associated with the decreased odds of preferring to feed a child with the specific type of food. Inverse association between increased duration of breastfeeding and preferring to feed a child with a specific type of food may be due to having better information on child feeding practice which has a great contribution to reduce child malnutrition. Similarly, the inverse relationship between an increased frequency of food buying and preferring to feed balanced diets for children could be associated with better access to infrastructure and food security, the major contributor for better child nourishment [31].

The main limitation of the study was that data were collected only from mothers/caretakers where involvement of both parents may better supplement the evidence.

## Conclusions

Despite the local availability of recommended diversity of food for the feeding of under-five children in the study are about 79% of mothers or caretakers of under-five children prefer to feed their children either family food (cooked for adult family) or a specific /monotonous/ type of food more than once a day having direct effect on reduction of dietary diversity, safety and acceptability of child food that intern might contribute for the increased and sustained prevalence of under-five malnutrition against efforts to reduce the magnitude in the study area and the nation at large. Therefore we recommended integration of child dietary diversity counseling session for mothers visiting health institution for ANC, PNC and immunization services and health professionals with IMNCI care and treatment guidelines.

## Additional files

Additional file 1: English questionnaire. This questionnaire was developed by the authors to assess parent’s food preference and its implication for child malnutrition in the study area. It has five parts that assess the sociodemographic, child health characteristics, maternal health characteristics, child feeding practice, and parents food preference sections. (DOCX 48 kb)

Additional file 2: Informed consent form. Informed consent form was prepared and attached at the front page of the questionnaire for participants to read and indicate their agreement or refusal for participating in this study. (DOCX 12 kb)

## Data Availability

The datasets used and/or analyzed during the current study are available from the corresponding author on reasonable request.

## Abbreviations

ANC: Antenatal care
ARRR: Adjusted relative risk ratio
CRRR: Crude relative risk ratio
FAO: Food and Agricultural organization
HDSS: Health and Demographic Surveillance System
HIV/AIDS: Human immune virus/Acquired immunodeficiency syndrome
IQ: Intelligent Quotient
KM: Kilometer
MDG: Millennium Development Goal
PNC: Postnatal care
