# Supplementary material for: Parent’s food preference and its implication for child malnutrition in Dabat health and demographic surveillance system; community-based survey using multinomial logistic regression model: North West Ethiopia; December 2017
Source: BMC Pediatr. 2019 Sep 2;19:304. doi: 10.1186/s12887-019-1692-3 (PMC6717982; doi:10.1186/s12887-019-1692-3)
Supplement: Supplementary file 1 — English questionnaire. This questionnaire was developed by the authors to assess parent’s food preference and its implication for child malnutrition in the study area. It has five parts that assess the sociodemographic, child health characteristics, maternal health characteristics, child feeding practice, and parents food preference sections. (DOCX 48 kb) [file 12887_2019_1692_MOESM1_ESM.docx]

## English questionnaire

Part 1. Sociodemographic information

- 1. **IDENTIFICATION**

Name of residential unit/kebele __________________

House number /code __________________

Date and time of data collection _________/time__________

- 1. **HOUSEHOLD information**

Now, I will like to have some information about members of your household

|  | Please, give me the names of all members of your household | Educational Level | | Age in Years | | Sex | Occupation | Relationship to HH Head (check below) |
| --- | --- | --- | --- | --- | --- | --- | --- | --- |
| 101 |  |  |  |  |  |  |  |  |
| 102. |  |  |  |  |  |  |  |  |
| 103. |  |  |  |  |  |  |  |  |
| 104 |  |  |  |  |  |  |  |  |
| 105. |  |  |  |  |  |  |  |  |
| 106. |  |  |  |  |  |  |  |  |
| 107. |  |  |  |  |  |  |  |  |
| 108. |  |  |  |  |  |  |  |  |

CODES FOR RELATIONSHIP TO HOUSEHOLD HEAD

01-(Head) 02 - (Spouse) 03 – (Son) 04 – (Daughter) 05 – (Daughter-in-law) 06 – Grandchild 07 – (Mother-in-law) 08 – (Sister) 09 – (Adopted/Foster child/Stepchild) 10 – (Not related) 88 – (Don’t know)

CODES FOR EDUCATIONAL LEVEL

00 - No education

01 - Primary

02 - Middle/JSS

03 - SSS/Secondary

04 - Higher

**CODES FOR SEX**

1. Male
2. Female

| **Part2 Child characteristics**   \|  \|  \| **Responses** \| \| \| **Skip to /remarks** \| \| \| --- \| --- \| --- \| --- \| --- \| --- \| --- \| \| 201 \| Child’s age \| _________in months \| \| \|  \| \| \| 202 \| Child’s sex \| 1. Male 2. Female \| \| \|  \| \| \| 203 \| Birth order of child’s \| ______________th \| \| \|  \| \| \| 204 \| Place of delivery \| 1. Home 2. Health facility \| \| \|  \| \| \| 205 \| Gestational age at birth \| 1. Less than 9 months 2. At 9 months 3. Greater than 9 months   99. Do not know /not sure. \| \| \|  \| \| \| 206 \| Was your child weighted at birth \| 1. Yes 2. No \| \| \| If no skip to 208. \| \| \| 207 \| If **YES** how much was the weight \| 1. Less than 2.5kg 2. 2.5- 4 kg 3. Greater 4 kg   99. I do not know. \| \| \|  \| \| \| 208 \| Types of birth \| 1. Single 2. Multiple /twines \| \| \|  \| \| \| 209 \| Did the birth of this child planed (Wanted) \| 1. Yes 2. No \| \| \|  \| \| \| 210 \| Did the child ever been immunized \| 1. Yes 2. No \| \| \| If no skip to 213 \| \| \| 211 \| vaccines received (from card or if no card ask for recall) \| 1. BCG only (see scar) 2. DPT-Hb-HBV(number of dose____) 3. Measles 4. No card found \| \| \|  \| \| \| 212 \| Vit.A supplementation in the past six months \| 1. Yes 2. No   99. do not know/not sure \| \| \|  \| \| \| 213 \| What do you think is the frequent health problem to the child? \| ____________diseases  99.Do not know \| \| \|  \| \| \| 214 \| Has the child had diarrhea in the last two weeks \| 1. Yes 2. No   99. Do not know /not sure \| \| \| If no skip 216 \| \| \| 215. \| If YES how frequent in a year? \| 1. Once 2. Twice 3. 3-4 times 4. More than five \| \| \|  \| \| \| 216 \| Has the child been ill with fever at any time in the last two weeks? \| 1. Yes 2. No   99. Do not know /not sure \| \| \|  \| \| \| 217 \| Presence of respiratory disease in the last two weeks \| 1. Yes 2. No   99. Do not know /not sure \| \| \|  \| \| \| 218 \| Presence of edema on child (Observe) \| 1. Yes 2. No \| \| \|  \| \| \| **Part. CHILD FEEDING AND CARING PRACTICES** \| \| \| \| \| \| \| \| 301 \| Did you ever breast feed the child (NAME)? \| \| 1. Yes 2. No \| \| \| If YES skip to 304 \| \| 302 \| reason for not breast feeding \| \| Reason________________ \| \| \|  \| \| 303 \| How long after birth did you first breast feed the child \| \| 1. Immediately 2. After ______hours (if less than 24 hours ) 3. After ____days   99. do not know /not sure \| \| \|  \| \| 304 \| Did you give the child (NAME) food or fluid immediately after birth before giving breast? \| \| 1. Yes 2. No \| \| \| If NO skip to 306 \| \| 305 \| If YES, what did you gave him /her \| \| 1. Water 2. Butter 3. Milk   99. Other specify \| \| \|  \| \| 306 \| Did you squeeze out and throw the first breast milk \| \| 1. Yes 2. No \| \| \|  \| \| 307 \| Are you still breasting feeding? \| \| 1. Yes 2. No \| \| \|  \| \| 308 \| How many times in last 24 hours did you breast fed \| \| __________times \| \| \|  \| \| 309 \| Do you breast feed in the night? \| \| 1. Yes 2. No \| \| \|  \| \| 310 \| Did you give the child additional food or fluid other than breast milk with in the past 48 hours? \| \| 1. Yes 2. No \| \| \|  \| \| 311 \| If YES, what ingredients you gave?  (More than one answer is possible) \| \| 1. Cow’s milk 2. Atmite 3. SSF made of cereal 4. SSF made of cereal combined with other product   96. Other (specify) \| \| \|  \| \| 312 \| How many times would give this food in last 24 hours \| \| _______times \| \| \|  \| \| 313 \| At what age did you start feeding other additional food? \| \| 1. Before six months 2. At six months 3. After six months   99.do not know /not sure \| \| \|  \| \| 314 \| What do you use to feed the child? \| \| 1. Bottle 2. Spoon 3. Cup \| \| \|  \| \| 315 \| How long did you breast feed the Child (NAME)? \| \| ____________months  99. Do not know /not sure \| \| \|  \| \| 316 \| For how many months did you exclusively breast fed the child? \| \| ____________months  99. Do not know/ not sure \| \| \|  \| \| 317 \| Who is usually taking care of the baby feeding \| \| 1. Mother 2. Sister 3. Grand mother   96. other specify \| \| \|  \| \| 318 \| How do you usually prepare food for children under five years age \| \| 1. Separately 2. Together with adult \| \| \|  \| \| 319 \| How do you give food for the child? \| \| 1. with adult and other older children 2. Separately 3. After adult and other older children 4. Before adult and other older children. \| \| \|  \| \| 320 \| During the illness has the child feeding practices changed? \| \| 1. Yes 2. No \| \| \| If NO skip to 322 \| \| 321 \| How could the practices changed \| \| 1. Feeding only breast milk 2. Providing additional food or fluid \| \| \|  \| \| 322 \| How did you usually treat your child when he/she get sick \| \| 1. Usually home treatment 2. Taking to traditional healers 3. Taking to Health institution/hospital \| \| \|  \| \| 323 \| Have you ever take your child to health Institution for sickness? \| \| 1. Yes 2. No \| \| \| If No skip to part 4 \| \| 524 \| How many times have you ever taken the child to health institution getting sick? \| \| __________times  99. do not know /not sure \| \| \|  \| \| **Part 4: MATERNAL CHARACTERISTICS** \| \| \| \| \| \| \| \| 401 \| Mother’s age in years \| \| ______completed year \|  \| \| \| \| 402 \| Age at first birth \| \| ______years \|  \| \| \| \| 403 \| Age when the youngest child was born \| \| ______years \|  \| \| \| \| 404 \| Total number of children ever born? \| \| In number_______ \|  \| \| \| \| 405 \| During pregnancy or lactation, did you  Consume extra food? (the child under the study) \| \| 1. Yes 2. No \|  \| \| \| \| 406 \| Health status during the pregnancy \| \| 1. Good 2. Not good /sick \|  \| \| \| \| 407 \| Did you visit health facility for ANC during pregnancy? \| \| 1. Yes 2. No \|  \| \| \| \| 408 \| Do you know about family planning? \| \| 1. yes 2. No \| If NO skip to 411 \| \| \| \| 409 \| Have you ever used family planning methods? \| \| 1. Yes 2. No \| If NO skip to 411 \| \| \| \| 410 \| Are you using it now? \| \| 1. Yes 2. No \|  \| \| \| \| 411 \| When do you usually wash your hands? \| \| 1. After latrine use 2. Before preparing food 3. Before serving food 4. After cleaning child feces \| More than one answer is possible \| \| \| \| 412 \| How do you wash your hand? \| \| 1. Using water only 2. Using soap some times 3. Using soap always 4. Using ash some times \|  \| \| \| \| 413 \| What do you think your child physically looks? \| \| 1. Thin 2. short 3. Big 4. Small \|  \| \| \| \| 414 \| For how long do you think should a child exclusively be breast-fed? \| \| In Months ______ \|  \| \| \| \|  \| \| \| \| \| \| \| | | |
| --- | --- | --- | --- | --- | --- | --- | --- | --- | --- | --- | --- | --- | --- | --- | --- | --- | --- | --- | --- | --- | --- | --- | --- | --- | --- | --- | --- | --- | --- | --- | --- | --- | --- | --- | --- | --- | --- | --- | --- | --- | --- | --- | --- | --- | --- | --- | --- | --- | --- | --- | --- | --- | --- | --- | --- | --- | --- | --- | --- | --- | --- | --- | --- | --- | --- | --- | --- | --- | --- | --- | --- | --- | --- | --- | --- | --- | --- | --- | --- | --- | --- | --- | --- | --- | --- | --- | --- | --- | --- | --- | --- | --- | --- | --- | --- | --- | --- | --- | --- | --- | --- | --- | --- | --- | --- | --- | --- | --- | --- | --- | --- | --- | --- | --- | --- | --- | --- | --- | --- | --- | --- | --- | --- | --- | --- | --- | --- | --- | --- | --- | --- | --- | --- | --- | --- | --- | --- | --- | --- | --- | --- | --- | --- | --- | --- | --- | --- | --- | --- | --- | --- | --- | --- | --- | --- | --- | --- | --- | --- | --- | --- | --- | --- | --- | --- | --- | --- | --- | --- | --- | --- | --- | --- | --- | --- | --- | --- | --- | --- | --- | --- | --- | --- | --- | --- | --- | --- | --- | --- | --- | --- | --- | --- | --- | --- | --- | --- | --- | --- | --- | --- | --- | --- | --- | --- | --- | --- | --- | --- | --- | --- | --- | --- | --- | --- | --- | --- | --- | --- | --- | --- | --- | --- | --- | --- | --- | --- | --- | --- | --- | --- | --- | --- | --- | --- | --- | --- | --- | --- | --- | --- | --- | --- | --- | --- | --- | --- | --- | --- | --- | --- | --- | --- | --- | --- | --- | --- | --- | --- | --- | --- | --- | --- | --- | --- | --- | --- | --- | --- | --- | --- | --- | --- | --- | --- | --- | --- | --- | --- | --- | --- | --- | --- | --- | --- | --- | --- | --- | --- | --- | --- | --- | --- | --- | --- | --- | --- | --- | --- | --- | --- | --- | --- | --- | --- | --- | --- | --- | --- | --- | --- | --- | --- | --- | --- | --- | --- | --- | --- | --- | --- | --- | --- | --- | --- | --- | --- | --- | --- | --- | --- | --- | --- | --- | --- | --- | --- | --- | --- | --- | --- | --- | --- | --- | --- | --- | --- | --- | --- | --- | --- | --- | --- | --- | --- | --- | --- | --- | --- | --- | --- | --- | --- | --- | --- | --- | --- | --- | --- | --- | --- | --- | --- | --- | --- | --- | --- | --- | --- | --- | --- | --- | --- | --- | --- | --- | --- | --- | --- | --- | --- | --- | --- | --- | --- | --- | --- | --- | --- | --- | --- | --- | --- | --- | --- | --- | --- | --- | --- | --- | --- | --- | --- | --- | --- | --- | --- | --- | --- | --- | --- | --- |
| **Part 5. parental preference of food for children to feed** | | |
| 501 | Food items available in locality | - Fruits and vegetables - Meat, chicken, fish, nuts and legumes - Eggs, milk, cheese and yogurt - Bread, cereals and grains - Others please list |
| 502 | Where the source the food items? | - Collecting from the garden or farm - Buying from the market |
| 503 | If you are buying from the market how often you buy? | - Daily - 2-3 times per week - Weekly - Fortnightly - Monthly - Quarterly - Never |
| 504 | How far do you travel one way to the nearest Km to this market | - Less than 1 Km - 1Km to 4Km - 5km t0 10 km - 11 to 20 km - 21km to 30 km - 31 to 40 km - 41 to 50 km - 51 or more km |
| 505 | How do you usually get to this market | - Walk - On back of animal( horse, donkey, mule ) - Public transport   Others ________________ |
| 506 | If never question 87 why? | - Too costly - Money spent on other activities - Not available in locality - Others specify |
| 507 | Which food items you preferred to feed for your child more than six months | - Fruits and vegetables - Meat, chicken, fish, nuts and legumes - Eggs, milk, cheese and yogurt - Bread, cereals and grains - Juice - Scanned foods /fast foods - Others |
| 508 | If you are given the choice which food item would you prefer to feed your child commonly | - Fruits and vegetables - Meat, chicken, fish, nuts and legumes - Eggs, milk, cheese and yogurt - Bread, cereals and grains - Juice - Scanned foods /fast foods - Others |
| 509 | If you are given the choice which food item would you prefer to feed your child always | - Fruits and vegetables - Meat, chicken, fish, nuts and legumes - Eggs, milk, cheese and yogurt - Bread, cereals and grains - Juice - Scanned foods /fast foods - Others |
| 510 | Why? | - It is to prepare - Child has good appetite - Less costly - Available - Others |
| 511 | If you are given the choice which food item would you prefer to feed your child not at all | - Fruits and vegetables - Meat, chicken, fish, nuts and legumes - Eggs, milk, cheese and yogurt - Bread, cereals and grains - Others |
| 512 | why? | - Children are not matured to digest extra food - No culturally permitted - If special diet is considered they may run wild   If other state_________________ |
