# Supplementary material for: Parent’s food preference and its implication for child malnutrition in Dabat health and demographic surveillance system; community-based survey using multinomial logistic regression model: North West Ethiopia; December 2017
Source: BMC Pediatr. 2019 Sep 2;19:304. doi: 10.1186/s12887-019-1692-3 (PMC6717982; doi:10.1186/s12887-019-1692-3)
Supplement: Supplementary file 2 — Informed consent form. Informed consent form was prepared and attached at the front page of the questionnaire for participants to read and indicate their agreement or refusal for participating in this study. (DOCX 12 kb) [file 12887_2019_1692_MOESM2_ESM.docx]

## **INFORMATION SHEET**

Dear participants this information is prepared for you to read and indicate your willingness or refusal to participate in this study. Please read the format and indicate your willingness or refusal by encircling numbers 1. I agree to participate or 2. I refuse to participate below.

**Title –** Parent’s food preference and its implication for child malnutrition in Dabat Health and Demographic Surveillance System; community-based survey using multinomial logistic regression model: North West Ethiopia,

**Purpose of the research**: The main aim of this study was to assess parent/caretakers food preference and its implication for child malnutrition in Dabat health and demographic site.
**Risk:** The study will take 30 minutes of your time during interview

**Benefits -** There are no known direct benefits to the study participant in this study.

“I have read this form or it has been read to me in the language I understand. I understand that I can discontinue the interview without any problem.”

1. I agree to participate
2. I refuse to participate if the participant agrees to participate, skip to the next page. If no, skip to the next participant by writing short reasons for refusal below. ………………………………………………………………………………………
3. Name of contact person : Nigusie Birhan Address: Mob +251-923-3938626

E-mail: nigusiebirhan@gmail.com

Thank you
